# Promoter Hypermethylation-Related Reduced Somatostatin Production Promotes Uncontrolled Cell Proliferation in Colorectal Cancer

**DOI:** 10.1371/journal.pone.0118332

**Published:** 2015-02-27

**Authors:** Katalin Leiszter, Ferenc Sipos, Orsolya Galamb, Tibor Krenács, Gábor Veres, Barna Wichmann, István Fűri, Alexandra Kalmár, Árpád V. Patai, Kinga Tóth, Gábor Valcz, Zsolt Tulassay, Béla Molnár

**Affiliations:** 1 2nd Department of Internal Medicine, Semmelweis University, Budapest, Hungary; 2 Molecular Medicine Research Unit, Hungarian Academy of Sciences, Budapest, Hungary; 3 1st Department of Pathology and Experimental Cancer Research, Semmelweis University, Budapest, Hungary; 4 1st Department of Pediatrics, Semmelweis University, Budapest, Hungary; Institut national de la santé et de la recherche médicale, FRANCE

## Abstract

**Background:**

Somatostatin (SST) has anti-proliferative and pro-apoptotic effects. Our aims were to analyze and compare the SST expression during normal aging and colorectal carcinogenesis at mRNA and protein levels. Furthermore, we tested the methylation status of *SST* in biopsy samples, and the cell growth inhibitory effect of the SST analogue octreotide in human colorectal adenocarcinoma cell line.

**Methods:**

Colonic samples were collected from healthy children (n1 = 6), healthy adults (n2 = 41) and colorectal cancer patients (CRCs) (n_3_ = 34) for *SST* mRNA expression analysis, using HGU133 Plus2.0 microarrays. Results were validated both on original (n_1_ = 6; n_2_ = 6; n_3_ = 6) and independent samples ((n_1_ = 6; n_2_ = 6; n_3_ = 6) by real-time PCR. SST expressing cells were detected by immunohistochemistry on colonic biopsy samples (n_1_ = 14; n_2_ = 20; n_3_ = 23). The effect of octreotide on cell growth was tested on Caco-2 cell line. SST methylation percentage in biopsy samples (n_1_ = 5; n_2_ = 5; n_3_ = 9) was defined using methylation-sensitive restriction enzyme digestion.

**Results:**

In case of normal aging SST mRNA expression did not alter, but decreased in cancer (p<0.05). The ratio of SST immunoreactive cells was significantly higher in children (0.70%±0.79%) compared to CRC (0%±0%) (p<0.05). Octreotide significantly increased the proportion of apoptotic Caco-2 cells. *SST* showed significantly higher methylation level in tumor samples (30.2%±11.6%) compared to healthy young individuals (3.5%±1.9%) (p<0.05).

**Conclusions:**

In cancerous colonic mucosa the reduced SST production may contribute to the uncontrolled cell proliferation. Our observation that in colon cancer cells octreotide significantly enhanced cell death and attenuated cell proliferation suggests that SST may act as a regulator of epithelial cell kinetics. The inhibition of SST expression in CRC can be epigenetically regulated by promoter hypermethylation.

## Introduction

Somatostatin (SST), a regulatory-inhibitory peptide, has remarkable effect on gastrointestinal function. It suppresses gastrointestinal motility and gall bladder contraction, inhibits gut exocrine secretion, regulates the intestinal nutrient absorption and blood flow, and decreases epithelial proliferation [1–5]. In addition SST inhibits hormone release (e.g. GH, TSH, insulin, gut hormones), serves as neurotransmitter and neuromodulator, and contributes to water balance [1,4,6]. Beyond these physiological endocrine and paracrine/autocrine effects, SST can directly inhibit cell proliferation and induce apoptosis via somatostatin receptor (SSTR) signaling [4,7]. Therefore, reduced somatostatin levels, which we found in a pilot study might have relevance in gastrointestinal tumorigenesis including colorectal cancer which is one of the leading causes of cancer-related death [8].

Somatostatin is mainly produced in the central and peripheral nervous system, in the endocrine pancreas and in the gut; furthermore minor SST secretion also has been proven in the thyroid, adrenals, submandibular glands, kidneys, prostate, placenta and the cells of the immune system. SST is synthesized from a large precursor molecule called preprosomatostatin (preproSST) that is processed enzymatically to mature products. Two bioactive peptide products of SST encoding gene are known, SST-14 and SST-28 [1]. SST-28 is synthesized in the intestinal mucosa, which is the largest peripheral source of the peptide and the major SST producing cells in the gastrointestinal tract are mucosal δ-cells in the intestinal epithelium, D-cells in the gastric antrum and pancreas, and intrinsic neurons in the myenteric and submucosal plexuses along the digestive tract [3]. Somatostatin release can be stimulated and inhibited by several hormones, neuropeptides, neurotransmitters, cytokines, growth factors and nutrients. For example, growth hormone-releasing hormone (GHRH), corticotropin-releasing hormone (CRH), neurotensin, bombesin, IL-1, IL-6 and TNF-α can stimulate the SST secretion in several tissues. On the other hand, the γ aminobutiric acid (GABA), opiates, TGF-β and leptin are potential inhibitors of SST release. Nutrients have tissue-specific effect on SST production, and gut SST secretion is triggered by luminal but not circulating nutrients [1].

The five known somatostatin receptors (SSTR1, SSTR2 /SSTR2A, SSTR2B/, SSTR3, SSTR4, SSTR5) encoded by five human genes are typical G-protein-coupled receptors (GPCR) with seven α-helical transmembrane segments. Upon binding of SST to its receptors, several cellular functions are modulated through multiple G-protein dependent signaling pathways. Different signaling pathways are activated depending on the receptor subtype and tissue localization. All SSTR subtypes inhibit adenylate cyclase and cAMP production, regulate protein phosphatases and activate G-protein regulated inward rectifier K^+^ channel (GIRK) family upon ligand binding [1,2,5,9,10].

The manifestation of antiproliferative and pro-apoptotic effects of SST on normal and tumorous cells depends on the type of ligand binding SSTR. SST and its analogs have indirect effects on cell growth by inhibiting the angiogenesis, modulating the immune system and inhibiting growth factors (e.g. IGF-1) and trophic hormones release. Furthermore, it can induce apoptosis, inhibit the cell cycle and modulate the impact of growth factors directly [11–14].

In our previous studies we have summarized the macroscopic, microscopic and molecular changes affecting the gastrointestinal tract, particular the large bowel during normal aging [15–17]. We have demonstrated that healthy juvenile colonic epithelium and colorectal cancer can be characterized with increased cell proliferation and decreased apoptosis compared to the healthy adult colonic mucosa. However, while cell proliferation is strictly regulated and well-balanced in children, it become uncontrolled in CRC. We have also revealed that alterations in mRNA expression during normal aging and colorectal carcinogenesis can be related to increased, but differently regulated cell growth [18]. The purpose of this study was to analyze local somatostatin production in human colonic epithelium during normal aging and colorectal carcinogenesis, both at mRNA and protein expression levels. We also investigated the effects of somatostatin analogue octreotide on human colorectal adenocarcinoma cell line (Caco-2). Since promoter hypermethylation can epigenetically alter gene transcription both during aging and carcinogenesis, we tested the methylation status of *SST* gene.

## Materials and Methods

### Patients and samples

After informed consent, colorectal biopsy samples were taken during routine endoscopic intervention at the 2nd Department of Internal Medicine and at the 1st Department of Paediatrics, Semmelweis University, Budapest, Hungary. Written informed consent was obtained in advance from all adult participants and from the next of kin, caretakers, or guardians on the behalf of the minors/children approved by the ethics committees. Altogether, 81 biopsy samples were analyzed in microarray analysis (6 healthy children, 41 healthy adults and 34 CRCs from adults) and 36 biopsy samples were involved in real-time PCR validation (12 healthy children, 12 healthy adults and 12 CRCs from adults). Fifty-seven tissue samples were analyzed by immunohistochemistry (14 healthy children, 20 healthy adults and 23 CRCs from adults) and 15 colonic biopsies were investigated using methylation-sensitive restriction enzyme methylation array analysis (5 healthy children, 5 healthy adults and 9 CRCs from adults). Seventy-five microarrays (containing the adult samples) had been hybridized earlier; their data files were used in previously published studies using different comparisons [19–21] and are available in the Gene Expression Omnibus database (series accession number: GSE10714 and GSE37364). The datasets of the newly hybridized 6 microarrays are registered on the GSE37267 serial accession number. Control children were referred to the outpatient clinic with constipation, rectal bleeding or chronic abdominal pain. Ileocolonoscopy was part of their diagnostic procedure (to exclude organic disease) and the biopsy specimens showed normal macroscopic appearance and histology. Every specimen was verified by histopathologists. For mRNA studies (microarray analysis, Taqman RT-PCR) and methylation array analysis, colonic samples were stored in RNALater Reagent (Qiagen Inc, Germantown, US) at −80°C before nucleic acid extraction. Colorectal biopsy samples were routinely fixed in formaldehyde and embedded in paraffin wax for immunohistochemistry experiments.

Ethical approvals (Nr.: 69/2008 and 202/2009) for this study were issued by the Regional and Institutional Committee of Science and Research Ethics of Semmelweis University (Budapest, Hungary). Detailed clinicopathological specification of the patient samples are summarized in *Table 1*.

**Table 1:** Subgroups of patients involved in Affymetrix microarray analysis, PCR validation, immunohistochemistry, and methylation array analysis with the number of samples and mean age values.

Number of patients participating in the study		
*Affymetrix microarray analysis*		
**Group**	**Number of samples (female/male)**	**Mean age ± SD (years)**
Children (Ch)	6 (2/4)	12.2 ± 5.3
Healthy adults (N)	41 (26/15)	53.0 ± 15.9
CRCs from adults (CRC)	34 (19/15)	68.5 ± 10.3
**Total patient number**	**81 (47/34)**	
*Taqman RT-PCR validation*		
**Group**	**Number of original samples (female/male)**	**Mean age ± SD (years)**
Children (Ch)	6 (2/4)	12.2 ± 5.3
Healthy adults (N)	6 (3/3)	57.7 ± 18.9
CRCs from adults (CRC)	6 (4/2)	69.0 ± 7.0
**Total patient number**	**18 (9/9)**	
**Group**	**Number of independent samples (female/male)**	**Mean age ± SD (years)**
Children (Ch)	6 (3/3)	5.5 ± 3.4
Healthy adults (N)	6 (3/3)	53.0 ± 16.9
CRCs from adults (CRC)	6 (1/5)	64.3 ± 10.8
**Total patient number**	**18 (7/11)**	
*Immunohistochemistry*		
**Group**	**Number of samples (female/male)**	**Mean age ± SD (years)**
Children (Ch)	14 (7/7)	9.86 ± 6.0
Healthy adults (N)	20 (11/9)	58.5 ± 14.0
CRCs from adults (CRC)	23 (13/10)	67.3 ± 10.5
**Total patient number**	**57 (31/26)**	
*Methylation array analysis*		
**Group**	**Number of samples (female/male)**	**Mean age ± SD (years)**
Children (Ch)	5 (2/3)	7.2 ± 6.3
Healthy adults (N)	5 (2/3)	56.8 ± 18.7
CRCs from adults (CRC)	9 (5/4)	65.8 ± 9.1
**Total patient number**	**19 (9/10)**	

### mRNA expression microarray analysis

According to the manufacturer’s instructions, total RNA was extracted using RNeasy Mini Kit (Qiagen Inc., Germantown, USA). The quantity of isolated RNA was characterized by measuring absorbance (NanoDrop ND-1000 Spectrophotometer, NanoDrop Technologies, Inc., Wilmington, USA) and the quality of isolated RNA was tested with capillary gel electrophoresis (2100 Bioanalyzer and RNA 6000 Pico Kit /Agilent Inc., Santa Clara, CA, USA/). According to the Affymetrix description, biotinylated cRNA probes were synthesized from 1 to 8 μg total RNA with RIN (RNA Integrity Number) between 7–10 and fragmented using the One-Cycle Target Labeling and Control Kit (http://www.affymetrix.com/support/downloads/manuals/expression_s2_manual.pdf). Ten micrograms of each fragmented cRNA sample was hybridized into HGU133 Plus2.0 array (Affymetrix, Santa Clara, CA, USA) at 45°C for 16 h. The microarrays were washed using Fluidics Station 450 device, and stained with antibody amplification staining method according to the manufacturer’s instructions. Fluorescent signals were detected by GeneChip Scanner 3000 (Affymetrix).

### TaqMan real-time polymerase chain reaction (RT-PCR) validation

TaqMan polymerase chain reaction was used to measure mRNA expression of SST encoding gene on original (6 histologically intact children, 6 histologically intact adult and 6 adult CRC samples) and independent sets of samples (6 histologically intact children, 6 histologically intact adult, 6 adult CRC samples). 18S ribosomal RNA (Hs03928990_g1*) and GAPDH (Hs03929097_g1*) were used as reference gene.

Total RNA extraction, quality and quantity controls were performed as described earlier. Using the High Capacity cDNA Reverse Transcription Kit with RNase Inhibitor, 1μg of total RNA per sample was reverse transcribed (Life Technologies, Carlsbad, CA, USA). The quality of cDNA was checked by SDHA real-time PCR (F. Hoffmann-La Roche Ltd., Basel, Switzerland). Then 16.7 ng cDNA template per sample was used for *SST* (Hs00174949_m1*) mRNA expression analysis with TaqMan Gene Expression Master Mix (Life Technologies). The measurements were carried out on LightCycler 480 (Roche) with Mono Color Hydrolysis Probe / UPL Probe detection format. After denaturation at 95°C for 10 min, 40 PCR cycles were carried out (amplification at 95°C for 15 sec, and at 60°C for 1 min).

### Immunohistochemistry for the detection of somatostatin producing cells

Cores of 2 mm diameter were collected from selected areas of formalin-fixed, paraffin-embedded tissue blocks prepared from 20 normal colon samples and 23 colorectal cancers of adult patients and inserted into tissue microarray (TMA) recipient blocks. Furthermore, 14 histologically intact colonic biopsy samples from children were also examined on protein level. 5 μm thick tissue sections were cut from TMA blocks and from biopsy samples, and were immunostained. Endogenous peroxidase blocking (0.5% hydrogen peroxide and methanol mixture, 30 min, room temperature) and antigen retrieval (Target Retrieval Solution, Dako, Glostrup, Denmark, code: S1699, in pH 6 buffer, performed in a microwave at 900 W for 10 min and at 370 W for 40 min) were carried out on dewaxed samples. Non-specific blocking with 1% bovine serum albumin was applied. Immunohistochemical detection of somatostatin was carried out in a humidified chamber using rabbit anti-human polyclonal antibody (1:50 dilution, overnight, Thermo Scientific, California, USA, code: RB-038-A). EnVision + HRP system (Labeled Polymer Anti-Rabbit, 40 min, Dako, code: K4003) and diaminobenzidine—hydrogen peroxidase—chromogen—substrate system (Cytomation Liquid DAB + Substrate Chromogen System, 10 min, Dako, code: K3468) were used for signal conversion. Finally, hematoxylin co-staining was carried out.

### Counting of somatostatin producing cells on digital slides

Stained biopsy samples and tissue microarray (TMA) slides were digitalized with a high resolution digital scanner (Pannoramic Scan, 3DHISTECH Ltd. Budapest, Hungary) using multilayer fluorescent scanning with a high numeric aperture (0.8) x20 objective lens and a high dynamic range AxioCam Mrm Rev.3 black-and-white camera connected to the scanner. Digital slides were accessed through a computer monitor and analyzed using the Pannoramic Viewer software (version 1.11.43.0). The Marker Counter software module resulting in permanent annotations on the counted cells was used to estimate the relative ratio SST producing and other epithelial cells. Epithelial SST positivity appeared all as strong brown cytoplasmic labeling in the slides. Depending on sample size, 500–1000 epithelial cells were counted in longitudinal well-oriented crypts.

### Treatment of Caco-2 cells with somatostatin analogue octreotide and measurement of Sub-G1 population using flow cytometry

**Cell culture and treatment.** Cell culture experiment was maintained in a specific pathogen cell culture laboratory. Caco-2 human epithelial adenocarcinoma cell line was obtained from DSMZ (Braunschweig, Germany; Cat. No. ACC-169). Cells were grown to confluence in MEM medium (Sigma-Aldrich, St. Louis, USA; Cat. No. M 2279), supplemented with 20% Fetal Bovine Serum (Sigma-Aldrich, St. Louis, USA; Cat. No. F 24429) and 160 μg/ml gentamycin (Sandoz GmbH, Schaftenau, Austria). Subsequently, 70.000 Caco-2 cells were settled for l well to a 24-well treatment plate in MEM supplemented with gentamycin and FCS. After 24 hours medium was changed: MEM was added with gentamycin and with 0.1, 1.0, 2.5, 5.0 and 10.0 nmol/l of octreotide (Sandoz GmbH) without FCS. Measurement was performed in triplicates for each concentration. After 24, 48 and 72 hour treatment the cells were harvested, two times washed in 0.5 ml sterile PBS and finally resuspended in 1.0 ml of ice cold 70% ethanol and stored at -20°C.

**Flow cytometry and Sub-G1 population detection.** Samples were centrifuged for 3 min at 1300 rpm, then the cells were resuspended in 300 μ1 of extraction buffer and 3 μl of RNAse (RNase A/T1 Mix, Thermo Fisher Scientific Baltics UAB, Vilnius, Lithuania) was added. After 15 min incubation at room temperature, 3 μl of propidium jodide (Sigma-Aldrich, St. Louis, USA; Cat. No. 81845) was added. The FACS measurement was performed on Becton Dickinson Immunocytometry Systems (Mountain View, California, USA, Serial No. 81313).

### Methylation-sensitive restriction enzyme methylation array analysis

Biopsy samples were homogenized in 2% sodium dodecyl sulphate for DNA extraction, and then incubated in Proteinase K digestion buffer (4 mg/mL) at 60°C for 16 hours. DNA extraction was performed with High Pure PCR Template Preparation Kit (Roche Applied Science, Penzberg, Germany) as instructed by the manufacturer. DNA was eluted in 2x100 μl RNase- and DNase-free water and stored at -20°C. DNA methylation profiles were examined using the EpiTect Methyl qPCR Array System (Qiagen, Hilden, Germany). The isolated DNA was incubated with either DNA methylation dependent or DNA sensitive restriction enzymes for 16 h at 37°C, then incubated at 65°C to halt methylase activity. Each 120 μL reaction contained 500 ng of genomic DNA. Following enzyme digestion, samples were analyzed by fluorescence-based, quantitative PCR using LightCycler 480 (Roche Diagnostics, Basel, Switzerland). PCR was performed at the following conditions: 95°C for 10 min, 40 cycles (97°C for 15 sec; and 72°C for 1 min—with real-time data acquisition). To ensure amplification of desired products, melting curve analysis was performed following the real-time PCR reaction. The melting curve data acquisition range was 65°–95°C, holding for 1 s at increments of 0.04°C for PCR product detection.

### Statistical evaluation

For mRNA expression profiling, the Affymetrix expression arrays were primarily pre-processed by GCRMA background correction method with quantile normalization and median polish summarization. The expression of *SST* gene among different sample groups was analyzed by ANOVA and post-test Tukey HSD. The datasets are available in the Gene Expression Omnibus databank (http://www.ncbi.nlm.nih.gov/geo/), series accession numbers: GSE10714, GSE37364 and GSE37267.

For real-time PCR validation of SST expression 12 samples were analyzed in the following groups: children, healthy/normal adults and adult CRCs. For normalization, 18S ribosomal RNA was used as housekeeping internal control and dCT values were represented on boxplots. Hence data was normal distributed for statistical analysis ANOVA test and Tukey HSD post-test were applied in order to determine significance. The following criteria were used: Fold change≤0.5 or Fold change≥2 and p-value<0.05.

For statistical analysis of SST immunostaining results the ANOVA test and Tukey HSD post-test were applied. In both methods significance criteria was p<0.05.

Mann-Whitney test was used to compare the proportion of Caco-2 cells in different cell cycle stages (Sub-G1, G1, S, G2 and M) of octreotide-treated groups and in the control group. p<0.05 was considered to be statistically significant.

The results of methylation array analysis were evaluated by ANOVA and Tukey HSD post-test to determine and compare the proportions of *SST* promoter methylation in the three sample groups.

For statistical analysis R 3.1.0 statistical environment was applied. Boxplots represents median and standard deviation data with outliers.

## Results

### Somatostatin expression alterations in hyperproliferative states of the colon

**Fig 1:**
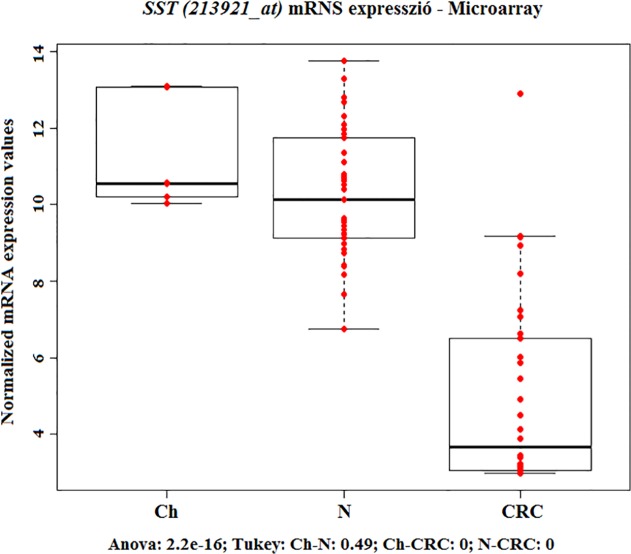
mRNA expression of *SST*—Microarray analysis. mRNA expression microarray analysis of somatostatin (SST) gene in microscopically normal colonic biopsy samples from children (Ch) and adults (N) and in colorectal cancer biopsy specimen (CRCs). Red dots are the normalized mRNA expression values, boxplots represent the median and standard deviation. *SST* expression significantly decreased in CRC.

Somatostatin mRNA expression levels in colorectal biopsy samples from healthy juvenile, adult and colorectal cancer were detected using 213921_at Affymetrix probeset ID (http://www.affymetrix.com/analysis/index.affx). mRNA expression of SST did not alter during normal aging as compared healthy juvenile (Ch) and adult (N) samples, however, gene expression significantly decreased in colorectal cancer (CRC) (p<0.05) compared to normal adult (N) samples *(Fig. 1)*.

**Fig 2:**
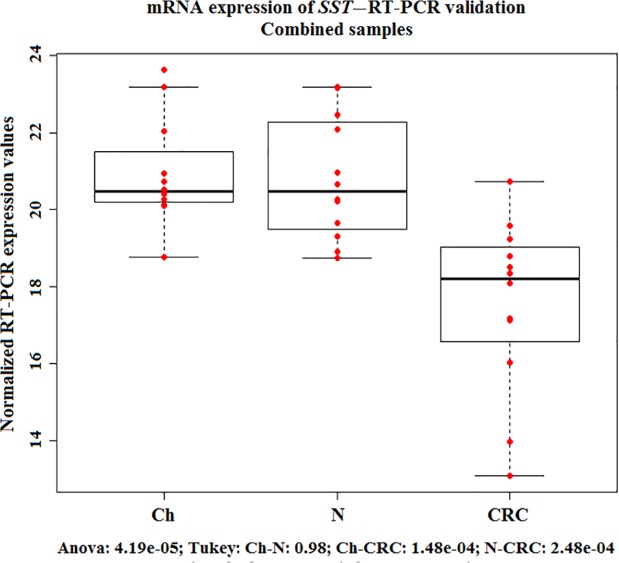
mRNA expression of *SST*—RT-PCR validation. Validation of mRNA expression changes of somatostatin (*SST*) gene in histological normal colonic biopsy samples from children (Ch) and adults (N) and in colorectal cancer biopsy specimen (CRCs) using real-time polymerase chain reaction (RT-PCR) on combined sample sets. Red dots are the normalized RT-PCR expression values, boxplots represent the median and standard deviation. RT-PCR analysis verified the significantly decreased *SST* expression in CRC.

**Fig 3:**
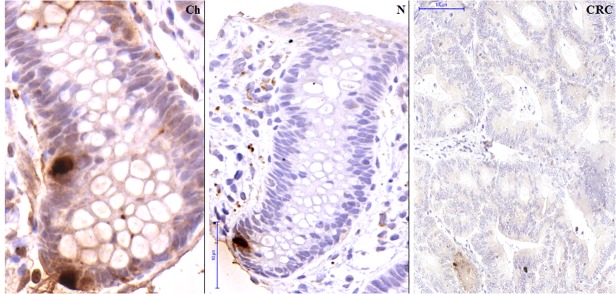
Immunohistochemistry for the detection of SST producing cells. Detection of SST producing cells (brown cytoplasm) during normal aging and in colorectal cancer (CRC). Images were taken with digital microscope: normal child tissue (Ch) (80-fold magnification), normal adult tissue (N) (55-fold magnification) and carcinoma (CRC) in adult (20-fold magnification). Only very low SST protein level could be detected in CRC samples.

**Fig 4:**
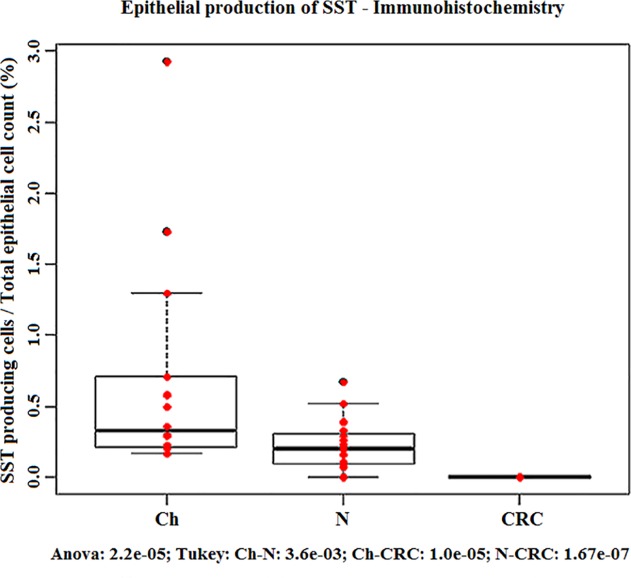
Epithelial production of SST—Immunohistochemistry. Detection of somatostatin (SST) producing cells in the human normal colonic epithelium from children (Ch) and from adults (N) and in colorectal cancers (CRCs). Red dots are the ratio of the SST producing cells compared to the total epithelial cell count (%); boxplots represent the median and standard deviation of the data. SST production significantly decreased in CRC.

Real-time PCR validation on the original, the independent and the combined sets of samples verified the significantly reduced *SST* expression in CRC (p<0.05) *(Fig. 2)*, and increased SST production in healthy samples, regardless of age. Immunohistochemical analysis confirmed the nearly absent somatostatin production in colorectal carcinoma samples as compared to young and adult healthy colonic mucosa on protein level (p<0.05) *(Fig. 3, 4)*.

### The anti-proliferative effects of somatostatin analogue on colorectal cancer cells

**Fig 5:**
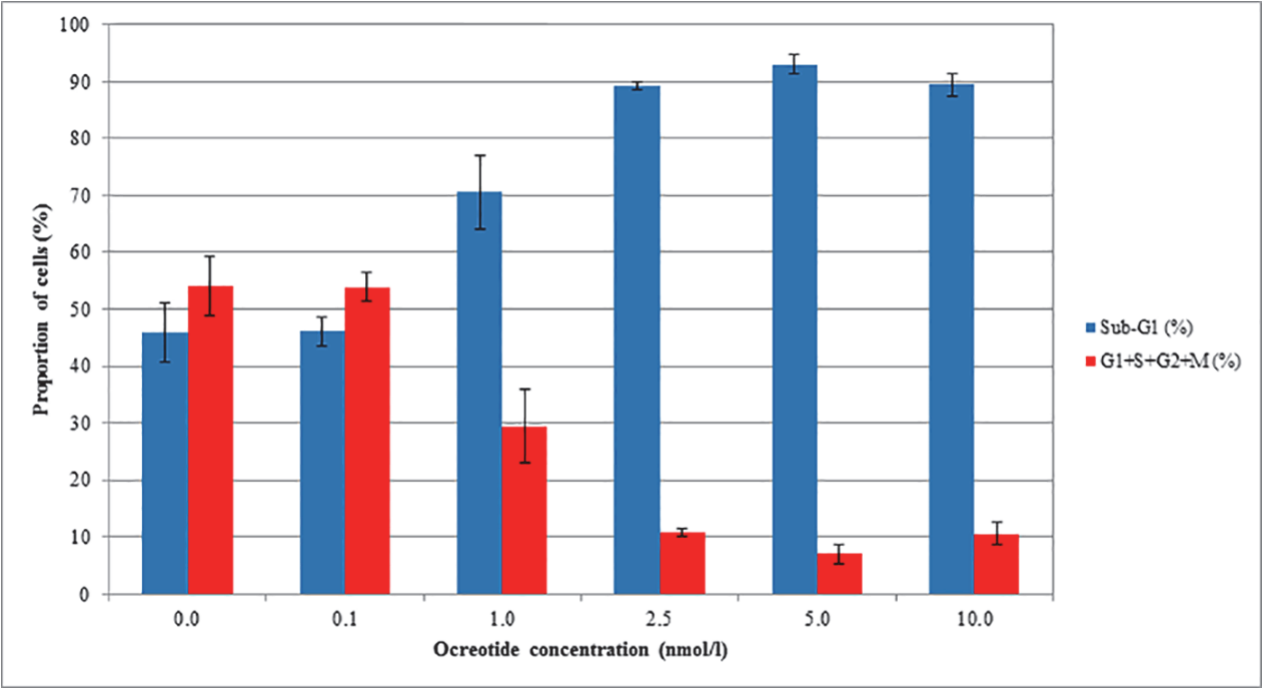
Caco-2 cell culture treatment with somatostatin analogue octreotide. Percentage of cells in control and octreotide-treated groups with average and standard deviation values. Blue columns represent the cells in Sub-G1 phase and the proportions of cells in G1+S+G2+M phase are illustrated by red columns. At higher concentrations than 0.1 nmol/l of added octreotide, the proportion of apoptotic Sub-G1 fraction was significantly higher (p<0.05) than in the control group, while the proportion of cells in other cell cycle phases (G1+S+G2+M) was significantly lower. The highest apoptotic fraction (Sub-G1) and the lowest (G1+S+G2+M) population were measured at 5.0 nmol/l octreotide concentration.

Somatostatin analogue octreotide was added to colorectal adenocarcinoma cells (Caco-2) in different concentrations. Significantly increased DNA fragmentation (Sub-G1 population) was measured by flow cytometry after 48 hours compared to the control cells, in a concentration-dependent manner. In cases of somatostatin analogue treatment at higher concentrations than 0.1 nmol/l, the proportion of apoptotic Sub-G1 fractions was significantly higher (p<0.05) than it was in the control group, while the proportion of cells in other cell cycle phases (G1, S, G2, M) was significantly lower. The highest apoptotic fraction (Sub-G1) and the lowest G1, S, G2, M population were measured at 5.0 nmol/l octreotide concentration. The flow cytometry results are summarized in *Table 2, Table 3* and *Fig. 5*.

**Table 2:** Distribution of Caco-2 cells in different stages of the cell cycle (average ± standard deviation), in the control and in the octreotide-treated groups.

Octreotide concentration (nmol/l)	Sub-G1 (%)	G1 (%)	S (%)	G2 (%)	M (%)
0.0	45.9 ± 5.2	32.2 ± 4.1	2.8 ± 1.5	13.0 ± 2.9	3.8 ± 0.4
0.1	46.1 ± 2.5	28.6 ± 0.7	2.7 ± 0.4	14.2 ± 2.2	5.7 ± 1.4
1.0	70.6 ± 6.4	11.9 ± 2.8	3.9 ± 2.7	7.3 ± 0.5	4.4 ± 1.1
2.5	89.2 ± 0.7	4.3 ± 0.8	1.2 ± 0.2	3.1 ± 1.2	1.5 ± 0.2
5.0	93.0 ± 1.7	3.9 ± 1.1	0.8 ± 0.4	1.5 ± 0.4	0.5 ± 0.2
10.0	89.5 ± 2.0	4.7 ± 0.8	1.1 ± 0.3	3.1 ± 0.5	0.5 ± 0.1

**Table 3:** P-values of Mann-Whitney rank correlation analysis.

Octreotide concentration (nmol/l)	Sub-G1	G1	S	G2	M
0.0 vs. 0.1	0.82	0.3827	0.8273	0.5127	**0.0495**
0.0 vs. 1.0	**0.0495**	**0.0495**	0.8274	**0.0495**	0.3827
0.0 vs. 2.5	**0.0495**	**0.0495**	**0.0495**	**0.0495**	**0.0495**
0.0 vs. 5.0	**0.0495**	**0.0495**	**0.0495**	**0.0495**	**0.0495**
0.0 vs. 10.0	**0.0495**	**0.0495**	**0.0495**	**0.0495**	**0.0495**

The distribution of Caco-2 cells in different cell cycle stages was correlated with the control group. The significant different p-values (p<0.05) are marked in bold.

### SST promoter DNA methylation alterations in hyperproliferative states of the colon

**Fig 6:**
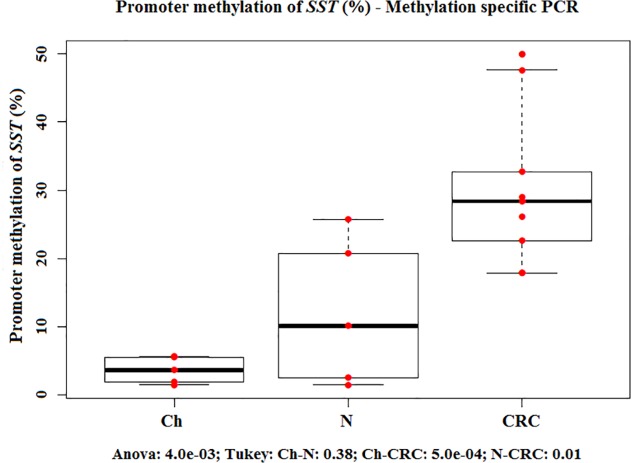
Promoter methylation of *SST* (%)—Methylation specific PCR. Evaluation of promoter methylation of *SST* gene in normal colorectal biopsy samples from children (Ch) and from adults (N) and in colorectal cancers (CRCs) using methylation-sensitive restriction enzyme methylation array analysis. Red dots are the promoter methylation values of *SST* (%); boxplots represent the median and standard deviation. The promoter methylation increases during normal aging and carcinogenesis, and the highest methylation rate was detected in the tumorous samples.

Promoter methylation of somatostatin gene increased during normal aging and carcinogenesis. The lowest *SST* promoter methylation was found in juvenile colonic epithelium (3.5%±1.9%) which can be characterized by controlled, increased cell proliferation. However, in colorectal cancer wherein increased cell growth is dysregulated, *SST* methylation was significantly higher (30.2%±11.6%) (p<0.05). The highest methylation status was detected in CRC *(Fig. 6)*.

## Discussion

Colorectal cancer is one of the most frequent malignant tumors with poor outcome in advanced cases. Its incidence is low under the age of 50, however, it increases rapidly in older age groups. In developed countries the median age at diagnosis is about 70 years [8] and in the majority of CRC cases is diagnosed over the age of 65 [22]. Therefore, age can be considered as a dominant risk factor for colorectal carcinogenesis [23]. Earlier we demonstrated that elevated cell proliferation is a common feature of colorectal epithelium both in infancy and in cancer with the significant difference in the control of cell proliferation in cancer [18]. In this study we demonstrated both at mRNA and protein levels that somatostatin expression does not differ significantly in elderly healthy colonic epithelium compared to juvenile samples, however, it is nearly absent in colorectal cancer. The promoter region of *SST* gene was found to be hypermethylated in CRC biopsies, which could be partly reversed by demethylation in cancer cell lines. Since treatment with the somatostatin analogue octreotide resulted in reduced cell proliferation and elevated apoptosis in a human colorectal adenocarcinoma cell line, our results suggested that the lack of local SST production may contribute to elevated and uncontrolled cell proliferation in CRC.

Gastrointestinal (GI) hormones (e.g. somatostatin, cholecystokinin (CCK), gastrin, bombesin (BBS)/gastrin-releasing peptide (GRP), neurotensin (NT), peptide YY (PYY), glucagon-like peptide 2 (GLP-2)) are secreted by endocrine cells, which are located in the intestinal mucosa and in the pancreas. These chemical messengers regulate intestinal and pancreatic secretion, digestion, absorption, motility and cell proliferation. Somatostatin is a regulatory-inhibitory peptide, which may be considered as a universal switch-off hormone and in addition it can inhibit cell growth in normal and neoplastic tissues. SST has endocrine and paracrine/autocrine effects, and can bind to its cell surface G protein-coupled receptors (SSTR1-5) initiating signal transduction pathways [24].

In this study we have found that the proportion of somatostatin producing cells is less than 1% in histologically normal young and adult colonic epithelium, and significantly reduced in tumorous samples. Previous immunohistochemical studies have demonstrated dual localization of somatostatin being both in endocrine D-cells and nerves in the large intestine [25]. Furthermore, *Parsons et al*. have also found relative low number of SST positive cells in the colonic epithelium [26]. In our workgroup *Galamb et al*. earlier identified genes (e.g. *AMN, PTGDR*) with decreasing expression during colorectal tumorigenesis [20,27]. Similarly to these markers, significantly decreased SST production may also contribute to colorectal cancer formation.

Due to the effects of SST on the GI tract, SST analogues can be applied effectively in non-neoplastic intestinal disorders such as acute variceal bleeding, pancreatic fistula, dumping syndrome, or in some cases of chronic diarrhea and intestinal pseudoobstruction [28].

Application of SST analogues is widespread regarding the diagnosis and therapy of neuroendocrine tumors [29–31]. Diagnosis of these tumors can be difficult and time consuming, that can worsen the chance of effective therapeutic intervention [30]. Radiolabelled somatostatin analogues can evince the localization of primary and metastatic tumors expressing somatostatin receptors that can not be detected with conventional imaging techniques due to their small size [32]. Furthermore, somatostatin and its derivates have several positive effects in the treatment of neuroendocrine neoplasms. The inhibition of hormonal hypersecretion can provide symptomatic relief and tumor shrinkage [7]. SST analogues can inhibit the production of GH and IGF, promoting factors of cancer growth [33]. They can inhibit angiogenesis [34,35], may play an immunemodulatory role [36,37], and can cause cell cycle arrest and induce apoptosis via somatostatin receptors [11,14]. SST analogues can be safer drugs for long-term use than cytotoxic chemotherapies, because they have fewer and milder side effects like gastrointestinal complaints, cholelithiasis and effects on glucose metabolism [30].

SST analogue scintigraphy can be applied for diagnosing immune-mediated disorders (e.g. lymphomas, granulomatous disease or rheumatoid arthritis) [38–41] and non-neuroendocrine tumors, as well [42]. There are several cell culture experiments, animal models and clinical studies investigating the positive effects of SST analogues in malignant tumors including lymphomas, breast cancer and prostate cancer [43–47].

In one clinical study the effect of octreotide was investigated in chemotherapy-refractory patients with gastric cancer. Significant improvement in survival was observed in the treated group, compared to the best-supportive care group [48]. The positive impact of SST analogue therapy on hepatocellular carcinoma (HCC) is not clear. In same cases octreotide administration improved the median survival compared untreated patients [49,50], but subsequent study in larger population did not show survival benefit for advanced HCC patients treated with long-acting octreotide compared with placebo [51]. In patients with ductal adenocarcinoma of the pancreas, tamoxifen treatment combined with octreotide increased the median survival times as compared untreated patients [52]. Summarizing other studies, objective beneficial effects of treatment with octreotide on tumor progression or survival were not found, however, in individual cases improvement in quality of life was observed [53].

Following the *in vitro* and *in vivo* studies clinical trials began in the 80s focusing on the possible role of somatostatin analogues in the treatment of colorectal cancers. *Savage et al*. examined the effects of a long-acting somatostatin analogue (SMS 201–995) in a pilot study on tumor growth in four patients with advanced colorectal cancer, but they did not find any evidence that the somatostatin analogue can alter the tumor growth [54]. In a phase II study, 16 patients with extensive CRC were treated with three daily subcutaneous injections of Sandostatin. Stable disease for 3–9 months was observed only in four cases, while other patients showed progressive disease. However, it is noteworthy that no adverse reactions were detected and most patients experienced temporary subjective improvements with a decrease in pain [55]. *Cascinu et al*. analyzed the anti-tumor effects of octreotide on patients with colorectal cancer refractory to chemotherapy as compared to the best supportive care. Authors concluded that the somatostatin analogue therapy confers survival benefit in advanced CRC [48]. Octreotide at a dose of 150 micrograms given three times daily subcutaneously was not effective in patients with asymptomatic, metastatic colorectal carcinoma in a randomized, controlled, double-blind, placebo-controlled phase III study [56].

Summarizing these data, preliminary clinical trials have reported disappointing results for somatostatin analogues in patients with CRC, but suggesting that the use of somatostatin analogues in incurable, end stage gastrointestinal cancers can reduce the unpleasant symptoms without any major side effects [57].

The diverse results of the studies found in literature may be caused by methodological alterations and clinical differences [58]. Among the five known somatostatin receptors primarily SSTR2 and SSTR5 mediate the antiproliferative effect of SST and its analogues. *Buscail et al*. demonstrated the loss of SSTR2 gene expression in advanced CRC and their respective metastases, which can also explain the inefficacy of somatostatin analogue therapy in some cases [59]. *Miller et al*. presented low affinity somatostatin receptor in normal and malignant colonic tissues. The possible explanation for the function of this receptor is that it may be involved in the locally produced SST regulation, in a paracrine or autocrine way, as opposed to a true endocrine mechanism. Therefore, low concentration of circulating somatostatin and its analogues can be functionally ineffective contrary to high local levels of SST within the colonic epithelium [60]. Based on these results, the antiproliferative effect of somatostatin analogue therapy in CRC can be essentially affected by the local epithelial concentration and the circulation half-life of the drug.

Forty-eight hours after addition of the somatostatin analogue octreotide, the proportion of apoptotic cells significantly increased, furthermore the proportion of cells in G1, S, G2 and M phase significantly decreased, in a concentration-dependent manner. Thus the octreotide treatment slowed down the growth of colorectal cancer cells. Previously *Dy et al*. also concluded that long-acting somatostatin analogue SMS 201.995 may have direct antitumor effects on human colon cancer cell lines [61]. Furthermore the results of *Colucci et al*. provided novel evidences that somatostatin can decrease COX-2 expression and enzyme function via activation SSTR3 or SSTR5 in human colon cancer cells, and suggests that these effects can contribute significantly to the inhibitory action of SST on cell growth [62]. Somatostatin analogues have also been extensively tested *in vitro* on other cultured cells derived from human, non-endocrine tumors with regard to their antiproliferative properties. Cell growth inhibition was detected in colorectal, gastric, pancreatic, breast, cervical, lung and prostatic cancer cell lines following administration of SST analogues [63].

DNA promoter methylation is an epigenetic gene silencing mechanism that regulates gene expression without changing the DNA sequence. Due to promoter hypermethylation transcription factors can not interact with DNA, diminishing gene expression. Many genetic alterations (e.g. point mutations, insertion-deletion mutations) can be associated with colorectal carcinogenesis, affecting for example *APC, KRAS, p53* and *SMAD4* genes. However, it has been shown in recent years that epigenetic changes also play an important role in the development of CRC [64,65]. In the present study we have demonstrated that promoter methylation of *SST* increases during normal aging and colorectal carcinogenesis and the highest methylation status was found in CRC. *SST* promoter hypermethylation was revealed in several gastrointestinal tumors in the past decade. *Mori et al*. similarly demonstrated the epigenetic silencing of somatostatin in CRC, and this suggests that inactivation of its growth suppression effect can be important step in colon tumorigenesis [66]. Increased methylation in the *SST* promoter region, causing gene silencing, was evinced in human esophageal carcinomas, and it also occurs early in Barrett-associated esophageal adenocarcinogenesis [67]. *SST* promoter methylation is a common event in human gastric cancer as well; it is connected with a decrease in SST protein and mRNA levels and associated with gastric carcinogens [68,69].

## Conclusions

Somatostatin is probably the most important naturally occurring anti-proliferative hormone. As we are aware, this is the first study to compare somatostatin expression in colorectal epithelium of healthy children and adults to that found in colorectal cancer samples both at mRNA and protein levels. Our results show that somatostatin production does not alter significantly during normal aging, but it is nearly absent in CRC. Earlier we observed increased proliferation and reduced apoptosis both in juvenile colonic epithelium and in CRC as compared to normal adult samples, but the control of cell growth was lost in CRC [18]. Our current results suggest that the significantly reduced epithelial somatostatin production may contribute to the accelerated and deregulated cell proliferation in CRC. This was supported by the inhibition of cancer cell growth along with the induction of apoptosis upon somatostatin analogue treatment. Reduced somatostatin levels were associated with promoter hypermethylation of *SST* gene as a potential explanation for the missing hormone in CRC. Further investigations are needed to test somatostatin analogues and demethylating compounds as potential therapeutic agents against sporadic colorectal cancer.
